# The status of licensed pharmacy workforce in Saudi Arabia: a 2030 economic vision perspective

**DOI:** 10.1186/s12960-018-0294-8

**Published:** 2018-06-28

**Authors:** Yazed AlRuthia, Mohammad A. Alsenaidy, Haitham K. Alrabiah, Abdullah AlMuhaisen, Mohammad Alshehri

**Affiliations:** 1 Department of Clinical Pharmacy, College of Pharmacy, King Saud University, P.O. Box 2454, Riyadh, 11451 Saudi Arabia; 2 Department of Pharmaceutics, College of Pharmacy, King Saud University, Riyadh, Saudi Arabia; 3 Department of Medicinal Chemistry, College of Pharmacy, King Saud University, Riyadh, Saudi Arabia; 4 College of Pharmacy, King Saud University, Riyadh, Saudi Arabia

**Keywords:** Pharmacist, Community pharmacy, Pharmacy workforce

## Abstract

**Background:**

The economy of Saudi Arabia is currently undergoing a major transformation which will have an impact on employment in the pharmacy sector. However, quantitative data characterizing the pharmacy workforce in the Kingdom are currently not available. Therefore, the aim of this study was to determine the current status of the licensed pharmacy workforce in the pharmacy field in Saudi Arabia.

**Methods:**

Descriptive statistics were performed on data from the Saudi Commission for Health Specialties (SCFHS) as of March 2017.

**Results:**

The labor market for pharmacists in Saudi Arabia is dominated by expatriates. Saudi nationals constitute less than 20% of the pharmacists employed in the Kingdom. The underemployment of Saudis is most evident in the largest sectors of the pharmacy field, namely, private health care establishments, community pharmacies, and pharmaceutical companies.

**Conclusion:**

There is an unmet need to train Saudi citizens as pharmacists and retain them in the workforce. Addressing this issue should become an important objective in Saudi Arabia’s Vision for 2030.

## Background

The Kingdom of Saudi Arabia is currently undergoing a strategic economic transformation, which poses unique challenges to the labor market, on the one hand, and to the education system, on the other. The field of pharmacy is not exempt from these changes. The number of pharmacy schools in Saudi Arabia is expanding rapidly: there was only one school in 2000, nine in 2008, 18 in 2010, and now, 27 in 2017. A majority of them are public schools [1, 2]. As a result, the number of pharmacists finishing education in public colleges also increased at a fast rate, that is, from 150–250 graduates in 2000 to 945 graduates in 2015 [3]. A study that compared the number of new students admitted to the pharmacy schools every year to the number of professionally active pharmacists in 13 Middle Eastern countries revealed that the ratio was 0.12 in 2008 [1]. This high ratio implies that if admitted students graduate from the study program and remain active in the profession, the number of pharmacists will continue to increase at a significant rate. In fact, some positive trends are already taking place. For example, the number of pharmacists in public hospitals has increased by 18%, from 1825 to 2154, in just a span of 2 years, that is, from 2010 to 2012 [4].

Despite this dynamic tendency, the number of certified Saudi pharmacists will remain too small to meet the high demand for this occupation in the foreseeable future [2, 3]. According to a report published by the World Health Organization in 2006, the number of pharmacists per 100 000 population is 20 in Saudi Arabia, 67 in Canada, 88 in the United States of America, and 169 in Egypt [1]. In another questionnaire-based cross-sectional study that explored the job preferences of pharmacy students who were in their final year at King Saud University, it was found that the majority of the students preferred to work in hospital settings rather than in the pharmacy industry [2]. In addition, it was reported that the estimated number of pharmacists required to satisfy the needs in all health care and industrial sectors in Saudi Arabia as of 2015 was 100 000 [2]. However, the most recent statistical yearbook of the Ministry of Health reported that there are only 22 829 pharmacists employed in different sectors in the Saudi Arabian economy [3]. This challenging situation can conceivably be addressed and remedied by the National Transformation Program introduced by the government of Saudi Arabia [5], known as Saudi Arabia’s Vision 2030. It aims at creating a condition for robust economic growth independent of traditional dependency on oil reserves. Currently, every workforce of the ministry in all regions of the Kingdom is being reviewed to determine staffing needs accurately. This action is expected to help in creating the optimal utilization of personnel, and this effort includes pharmacists, as well [4]. It will also help create many job opportunities for Saudi citizens, especially since the country has a relatively high unemployment rate among its youth [6]. Therefore, all the new regulations that are aimed to reform the labor market emphasize on replacing foreign workers with Saudi nationals [7]. These new regulations entail effective implementation of previously enacted governmental programs, such as “Nitaqat”, that incentivize businesses which employ Saudis with financial rewards such as paying part of the salary for all newly employed citizens in the first year of their employment [8]. However, such programs have somewhat failed in bringing the unemployment rate down, largely due to the perception among some private business owners that Saudis are more expensive to hire and are not as productive as their foreign counterparts [9, 10]. In addition, the Saudi market has many small businesses (< 10 employees) particularly in the community pharmacy sector, and they can go bankrupt if foreign workers in such businesses were abruptly and not gradually replaced with citizens, mainly due to the higher salaries owed to citizens [11, 12]. Thus, improving the quality of pharmacy education is imperative to ensure a smooth transition in implementing pharmacy workforce nationalization [13, 14].

As of today, three out of seven internationally accredited undergraduate pharmacy programs by the Accreditation Council of Pharmacy Education are in Saudi Arabia [15]. Furthermore, three out of five internationally accredited undergraduate pharmacy programs by the Canadian Council for Accreditation of Pharmacy Programs (CCAPP) are in Saudi Arabia [15]. The number of the Saudi colleges of pharmacy seeking accreditation from these international accrediting bodies is expected to increase over the next 5–10 years with the National Transformation Program [5]. In addition, the Medical Improvement Project of the Ministry of Health requires the presence of a specialized pharmacist that is trained to educate doctors and nurses about the optimal methods for prescribing medication in each hospital [4]. Therefore, the demand for hospital pharmacists with post-graduate residency training is increasing [16, 17]. The Saudi Commission for Health Specialties (SCFHS) has started general and specialized pharmacy residency programs with the first program having begun in 2001 [18]. Today, four general and three specialized pharmacy residency programs in four different health care institutions in the Kingdom are accredited by the American Society of Health-System Pharmacists, and the number is expected to rise [19]. Moreover, five different colleges of pharmacy are offering Master of Science in clinical pharmacy and three of them are also offering Master of Science programs in pharmaceutical sciences. However, only King Saud University is offering Doctor of Philosophy (PhD) programs in pharmaceutical sciences [20].

Barriers to pharmacy workforce nationalization are not limited to the quality of education alone, as cultural barriers also exist [21]. Although women represent the majority of the university graduates in Saudi Arabia, their unemployment rate is over 36% [22, 23]. However, with the new economic vision, women are empowered through different government initiatives to actively participate in the workforce, especially in the health care sector [5]. The success of the above-indicated programs is dependent on the availability of quantitative data characterizing the pharmacy workforce in the Kingdom of Saudi Arabia. However, such data are currently not available.

In order to practice pharmacy in Saudi Arabia, pharmacists and pharmacy technicians must be licensed by the SCFHS. This entails submitting all academic credentials which are at least a 5-year bachelor’s degree in pharmacy for pharmacists or a 2-year pharmacy technician associate’s degree for pharmacy technicians from an accredited academic institution by the Ministry of Education, as well as passing the licensure examination [24]. Therefore, the purpose of the present investigation was to determine, in quantitative terms, the current status of the licensed pharmacy workforce in the pharmacy field in Saudi Arabia. Specifically, we sought to ascertain the number of pharmacists and pharmacy technicians working in each of the following sectors: hospital pharmacy including governmental non-military health care settings, governmental military health care settings, and private health care settings, community pharmacies, academic institutions including private and public pharmacy colleges, and the pharmaceutical industry which includes national and international pharmaceutical companies. Moreover, we addressed the question of the participation of Saudis in the pharmaceutical workforce, the contribution of males and females to the pharmacy personnel, and their distribution among Saudi Arabian provinces. Classification of pharmacists according to the Saudi Commission for Health Specialties was addressed as well.

## Methods

This cross-sectional study utilized information collected by the SCFHS. The SCFHS is the legal body responsible for licensing all health care professionals in the Kingdom of Saudi Arabia. In order to practice in any health care setting, individuals with health care degrees must submit their academic credentials to the SCFHS. Once their degrees are verified, they will sit for their professional licensure examination, which they have to pass in order be licensed to practice in Saudi Arabia. Therefore, the SCFHS keeps the records of all licensed health care professionals in Saudi Arabia. The data were retrieved from the Department of Research and Statistics of the SCFHS and show the information of all licensed pharmacists as of March 2017.

For statistical evaluation of the data, Statistical Analysis Software (version 9.2, SAS Institute Inc., Cary, NC, USA) and Prizm (version 7.03, GraphPad Software Inc., La Jolla, CA, USA) were employed. The chi-square test for the comparison of two sets of values and Pearson’s test for correlation between two variables were used. *P* values of less than 0.05 were considered to be statistically significant.

## Results

The total number of subjects included in the database was 24 395.

As of March 2017, there were 24 395 licensed pharmacists and pharmacy technicians employed in the Kingdom of Saudi Arabia (Table 1**)**.

**Table 1:** The socio-demographic characteristics of the licensed pharmacy workforce in the Kingdom of Saudi Arabia

Characteristic^a^	Nationality		*P* value	Total (*N* = 24 395)
	Saudi (*N* = 4 531)	Non-Saudi (*N* = 19 864)		
Age (years ± SD)	37.46 ± 8.29	38.06 ± 8.87	< 0.001	37.97 ± 8.78
Sex				
Males, *n* (%)	3 182 (70.23)	17 594 (88.57)	< 0.001	20 776 (85.16)
Females, *n* (%)	1 349 (29.77)	2 270 (11.43)		3 619 (14.84)

^a^Data are expressed as *N* (%) and mean ± standard deviation (SD)

**Fig. 1:**
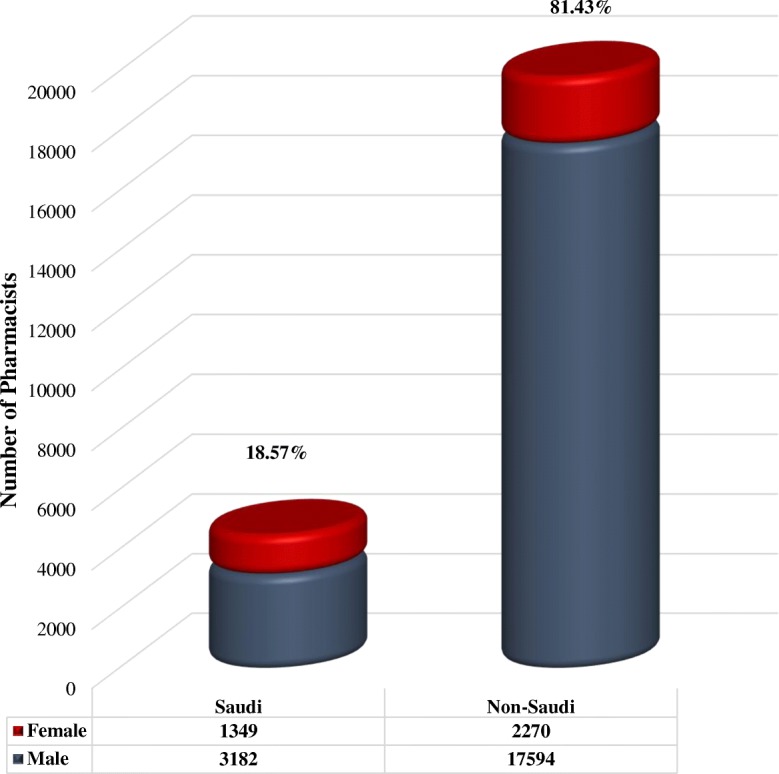
The percentage of Saudis in the pharmacy workforce of the Kingdom

A minority of them, 4531 (18.6%), are Saudi nationals, while a vast majority, 19 864 (81.4%), are non-Saudi citizens, representing 55 different nationalities. Among them, Egyptian citizens are the most numerous, with 13 907 pharmacists accounting for 57% of the workforce. Table 1 provides further the socio-demographic characteristics of pharmacists and pharmacy technicians in Saudi Arabia in detail. The average age of pharmacists and pharmacy technicians is 38 years, with a slightly (0.6 years) but significantly (*P* < 0.001) higher mean age of non-Saudi employees. The majority of them are male (85.16%) of which 15.32% are Saudis (*P* < 0.001 vs. non-Saudis). Out of 3619 licensed female pharmacists and pharmacy technicians, 37.28% are Saudis (*P* < 0.001 vs. non-Saudis). Thus, the number of males in the pharmacy workforce is 4.4-fold higher than the number of females. This ratio is markedly higher (7.8-fold) among non-Saudi than among Saudi pharmacists and pharmacy technicians (Fig. 1).

Seven health care-related sectors which provide jobs for pharmacists were introduced, namely, governmental non-military health care, governmental military health care, private health care, community pharmacies, academic institutions, the pharmaceutical industry, and unspecified facilities. The largest number of pharmacists, 8419, is employed in community pharmacies (Table 2**)**.

**Table 2:** Distribution of the licensed pharmacy workforce in the Kingdom across the pharmacy sectors

Sector^a^	Nationality		*P* value	Total *N* (%)
	Saudi *N* (%)	Non-Saudi *N* (%)		
Non-military governmental institutions	2 857 (63.05)	1 367(6.88)	< 0.0001	4 224 (17.32)
Military health care institutions	366 (8.08)	266 (1.34)		632 (2.59)
Private health care institutions	430 (9.49)	2 998 (15.09)		3 428 (14.05)
Community pharmacies	112 (2.47)	8 307 (41.82)		8 419 (34.51)
Pharmaceutical companies	504 (11.12)	6 392 (32.18)		6 896 (28.27)
Academic institutions	188 (4.15)	239 (1.20)		427 (1.75)
Unspecified	74 (1.64)	296 (1.49)		369 (1.51)
Total	4 531 (100)	19 864 (100)		24 395 (100)

^a^Data are expressed as *n* (%)

**Fig. 2:**
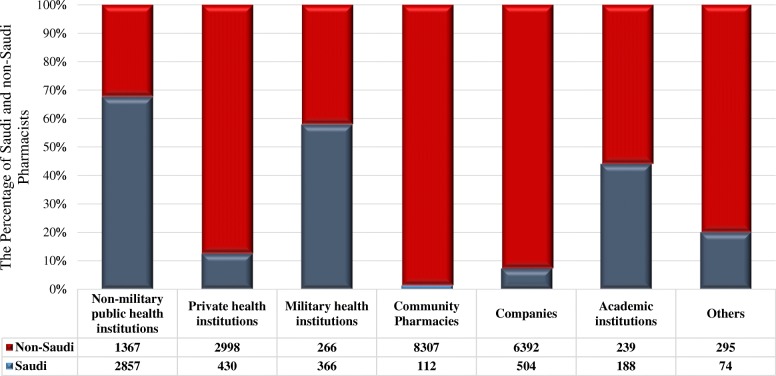
The number and percentage of Saudis and non-Saudis in different pharmacy sectors

They constitute 34.5% of the total pharmacy workforce. In this sector, 98.7% of workers are non-Saudi citizens. For every Saudi Arabian citizen working in a community pharmacy, there are 74 non-Saudi citizens employed. Similar circumstances exist in the second largest sector, the pharmaceutical industry. This branch of the economy employs 6896 workers, that is, 28.3% of the total number of pharmacists. In pharmaceutical companies, 92.7% of employees are non-Saudi citizens; for every citizen of Saudi Arabia working in this sector, there are 13 non-Saudi citizens employed. Private health care institutions, the fourth largest employer of pharmacists in Saudi Arabia (3428 employees; 14.0%), exhibit the same pattern regarding the nationality of pharmacists. In this sector, 87.5% of workers are non-Saudi citizens; for every single citizen working in the setting of private health care institutions, there are seven non-Saudi citizens employed (Fig. 2).

Governmental health care institutions, non-military and military combined, employ 4856 workers, that is, 20% of the pharmacy workforce in Saudi Arabia (Table 2). In contrast to the sectors analyzed in the preceding paragraphs, citizens of the Kingdom constitute a majority (66.3%) in these health care establishments (67.6% in the non-military and 57.9% in the military institutions). Finally, academic institutions, both public and private, employ 427 pharmacists (1.7% of the total pharmacy personnel). Saudis constitute 44% of this group of employees (Fig. 2).

Table 3 provides information on the number of Saudi and non-Saudi pharmacists and pharmacy technicians in the 13 provinces of Saudi Arabia.

**Table 3:** The distribution of Saudi and non-Saudi licensed pharmacy workforce across the administrative provinces of the Kingdom

Province^a^	Nationality		*P* value	Total *N* (%)
	Saudi *N* (%)	Non-Saudi *N* (%)		
Riyadh	1 879 (41.47)	6 816 (34.3)	< 0.0001	8 695 (35.64)
Makkah	863 (19.05)	5 237 (26.36)		6 100 (25.02)
Madinah	190 (4.19)	942 (4.74)		1 132 (4.64)
Qassim	174 (3.84)	763 (3.84)		937 (3.84)
Eastern Province	622 (13.73)	3 116 (15.69)		3 738 (15.32)
Asir	246 (5.43)	945 (4.76)		1 191 (4.88)
Tabuk	83 (1.83)	364 (1.83)		447 (1.83)
Ha’il	75 (1.66)	423 (2.13)		498 (2.04)
Northern Borders	51 (1.13)	135 (0.68)		186 (0.76)
Jizan	146 (3.22)	461 (2.32)		607 (2.49)
Najran	90 (1.99)	277 (1.39)		367 (1.50)
Bahah	56 (1.24)	182 (0.92)		238 (0.98)
Jawf	56 (1.24)	203 (1.02)		259 (1.06)
Total	4 531 (100)	19 864 (100)		24 395 (100)

^a^Data are expressed as *n* (%)

**Fig. 3:**
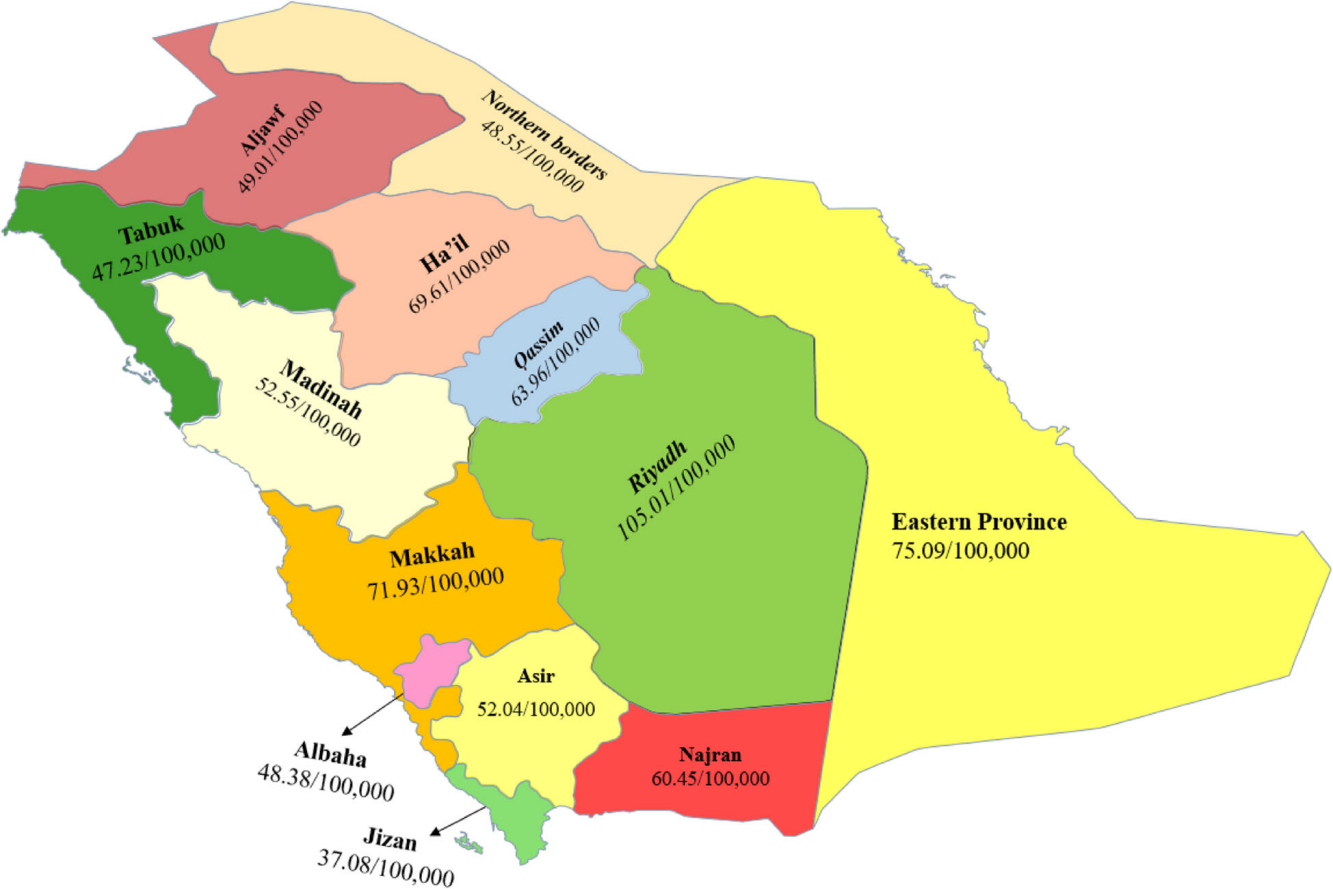
The number of pharmacists per 100 000 people in the provinces of the Kingdom

The fraction of Saudi pharmacists varies significantly, from 14.1% in the Makkah province to 27.4% in the Northern Borders. The distribution of the number of pharmacists per 100 000 population in these regions exhibits marked disparities (Fig. 3). It ranges from 37.08 in the Jizan province to 105.01 in the Riyadh province, with an overall average of 74.03 pharmacists per 100 000 population. Interestingly, with the exception of the cosmopolitan province of Riyadh, there is a robust negative correlation (Pearson’s correlation coefficient: − 0.69, *P* < 0.02) between the fraction of Saudi pharmacists and the number of pharmacists per 100 000 residents.

Table 4 lists professional categories of licensed workers, as defined by the SCFHS.

**Table 4:** The SCFHS classifications of the licensed pharmacy workforce in the Kingdom

Classification	Nationality		*P* value	Total *N* (%)
	Saudi *N* (%)	Non-Saudi *N* (%)		
Pharmacist	3 715 (81.99)	18 025 (90.74)	< 0.0001	21 740 (89.12)
Clinical pharmacist	66 (1.46)	8 (0.04)		74 (0.30)
Pharmacist one	421 (9.29)	130 (0.65)		551 (2.26)
Consultant pharmacist	116 (2.56)	32 (0.16)		148 (0.61)
Specialist	3 (0.07)	1 (0.01)		4 (0.02)
Pharmacy technician	181 (3.99)	305 (1.54)		486 (1.99)
Technician assistant	8 (0.18)	11 (0.06)		19 (0.08)
Specialty pharmacist	3 (0.07)	0 (0.00)		3 (0.01)
Non-practicing pharmacist	18 (0.40)	1 352 (6.81)		1 370 (5.62)
Total	4 531 (100)	19 864 (100)		24 395 (100)

Data are expressed as *n* (%)

The category of a pharmacist is the prevailing one with 21,740 employees. Saudi nationals constitute 17.1% of workers in this category, a value similar to their overall participation in the pharmacy workforce (18.9%). Specialized positions of clinical pharmacist, pharmacist one (with a Master of Science degree or equivalent), consultant pharmacist, specialist, and specialty pharmacist are filled mostly by Saudis; their participation varies from 75% in the pharmacist one category to 100% among specialty pharmacists. The contribution of Saudis to pharmacy technicians and technical assistants averages 40%. Finally, non-practicing pharmacists are almost exclusively non-Saudi nationals; they constitute 98.7% of this job category.

## Discussion

The analysis of data on the pharmacy workforce performed in the current study provides information that is crucial for the successful implementation of the goals set forth in Saudi Arabia’s Vision 2030. The most significant finding is that the Saudi nationals constitute less than 20% of the pharmacists employed in the Kingdom. The underemployment of Saudis is most apparent in the largest sectors of pharmacy institutions, namely, the private health care establishments, community pharmacies, and pharmaceutical companies. Moreover, the findings of this study indicate a significant increase in the number of pharmacists, when compared with previously published studies [1–3]. According to the World Health Organization Report of 2006, the number of pharmacists per 100 000 people in Saudi Arabia was 20 [1]. However, this study showed the number of pharmacists per 100 000 people in 2017 as 74.03 per 100 000 people. This number is higher than the number of pharmacists per 100 000 people in Canada (67) and lower than the number of pharmacist per 100 000 people in the United States (88) [1]. Further, the number of licensed pharmacists in Saudi Arabia that was reported in this study (23 890 pharmacists) contradicts the 100 000 pharmacists needed to replace all foreign pharmacists in the Kingdom that was reported in another study that was only based on predictions [2].

Traditionally, the labor market in Saudi Arabia was highly dependent on foreign workers. A leading cause for this condition was the high demand for workers in the oil industry and in large infrastructure projects [6]. The latter does not ensure continuity of employment, making them less attractive for Saudi nationals who are looking for stable jobs. It may come, however, as a surprise that a similar over-reliance on foreign workers prevails in the pharmacy field.

The regulations to be implemented under the new labor law emphasize the relevance of increasing the participation of Saudi nationals in the country’s workforce and the gradual reduction of the number of foreign workers [7]. The presence of the negative correlation between the fraction of Saudi pharmacists and the number of pharmacists per 100 000 population, reported in this study (Fig. 3), indicates that the immigrant workforce does not target the underserved areas. This notion further emphasizes the need to train Saudi Arabians as pharmacists and retain them in the workforce; this should become an important objective of Saudi Arabia’s Vision 2030.

The “Saudization” policy currently enforced in the Kingdom aims at replacing expatriates by local residents. The “Nitaqat” rules were introduced to increase the effectiveness of the “Saudization” policy. They involve evaluating private sector establishments by the percentage of Saudis in their workforce in order to reward the establishments with government-provided benefits [8]. Although locals and expatriates are equally competent and employable, existing limited research indicates that private employers view job applicants who are expatriates favorably, mostly due to the perception that their work ethics are superior [6, 9].

In contrast to common belief, non-Saudi employees do not have better education than their Saudi counterparts [8, 10]. This fact defies the rationale of the notion that non-Saudis are preferentially recruited because of their higher levels of education. Whether this equivalency of education between Saudis and non-Saudis is present also among pharmacy workers is an important question that remains to be answered urgently.

Nitaqat, a program of the Saudi Ministry of Labor aiming at the increase of employment of Saudi citizens, might provide a partial solution to the problem of excessive employment of expatriates. However, establishments with less than 10 employees are exempt from this program, and essentially all community pharmacies—collectively the largest employers of pharmacists—are included in the category of small establishments [11]. Moreover, Saudization has to be applied to the pharmacy sector with extreme caution, as underscored by recent closures of reportedly 200 000 businesses as a result of imposing quotas for Saudi employees [12]. Arguably, the recurrence of this phenomenon in case of pharmacies would have disastrous consequences for the residents in Saudi Arabia. Thus, exceptional care must be exercised in Saudization of the pharmacy workforce.

It is also expected that the Nitaqat approach will have a positive impact on the employment of women [8]. Whether this will also include female pharmacists or not remains to be seen. The current participation of women in the Pharmacy profession, 14.8% (Table 1), is comparable to that in the overall economy, 15.2% [21]. However, the unemployment among women willing to work increases at a high rate, from 16% in 2000 to 36% in 2012 [22]. This may reflect the reluctance of business owners to deal with cultural barriers associated with employing women in a manner compliant with the Islamic traditions and Saudi culture [6, 15]. If this is the case, additional incentives may have to be included in the program of Saudization of the pharmacy workforce.

The education of a new cadre of Saudi pharmacists is essential for the success of Saudi Arabia’s Vision 2030, as related to the pharmacy field. The consensus among experts, including the faculty of pharmacy schools and managerial personnel is that education of pharmacists in the country needs improvement and that educational outcome measures for pharmacy programs are required at the national level [13]. Although the majority of clinical pharmacists, specialist pharmacists, and consultant pharmacists are Saudis, their number is small compared to the number of pharmacists. This suggests that the number of pharmacists with post-graduate training needs to be increased in order to meet the needs of the residents in Saudi Arabia [13, 16]. This can be achieved through increasing the number of graduate programs in pharmacy, such as residency, fellowship, Master of Science, and Doctor of Philosophy programs in different fields of pharmacy [16, 20]. Another vital issue, particularly given the perceived lack of experience of Saudi candidates for employment [6, 9], is the need to provide pharmacy practice training for graduates of pharmacy colleges [17]. Arguably, this will improve the attractiveness of Saudi pharmacists when competing for employment with expatriates.

Another underappreciated issue is the involvement of pharmacists in pharmaceutical research. Among the Middle Eastern countries, Saudi Arabia has the highest number of pharmacists working in hospitals. However, their output in terms of practice-based research is limited [25]. Additionally, the need for conversion from an inpatient pharmacy model to an ambulatory care service system provided by the community or retail pharmacies has been voiced [14], and this transformation will require additional numbers of highly qualified pharmacists.

It may also be noted that the data maintained by the Department of Research and Statistics of the SCFHS employ different professional classifications of pharmacists than what is stated in the SCFHS’ own guidelines for classification of health care practitioners [24]. While this can cause inaccuracies in the records of the Commission, we do not perceive that it may have materially affected our analysis and conclusions. Another potential limitation of our study is that there is no record of unlicensed pharmacists, if any, who might be employed in non-health sectors, such as marketing and consultancy firms, in which a professional license is not required. However, this specific issue lies beyond the scope of the present investigation. Moreover, this was a cross-sectional study in which the findings are only valid as of March 2017.

## Conclusion

This study confirmed the under-representation of the Saudi pharmacists in the pharmacy workforce in the Kingdom of Saudi Arabia. It also sheds light on the importance of investing in graduate pharmacy education, as well as improving the quality of the undergraduate pharmacy programs, to enable new graduates to fill the job positions of foreign pharmacists in different sectors, especially the community pharmacy sector. Overall, the data presented in this report provides a snapshot of the situation as of March 2017, and more research will be required in the coming years to monitor the effectiveness of the transition to the new economic paradigm.

## Abbreviations

AA: Abdullah AlMuhaisen
CCAPP: Canadian Council for Accreditation of Pharmacy Programs
HA: Haitham Alrabiah
MA: Mohammad Alshehri
MAA: Mohammad A. Alsenaidy
PhD: Doctor of Philosophy
SCFHS: Saudi Commission for Health Specialties
YA: Yazed AlRuthia
